# Polymer-Based Chemicapacitive Hybrid Sensor Array for Improved Selectivity in e-Nose Systems

**DOI:** 10.3390/s25134130

**Published:** 2025-07-02

**Authors:** Pavithra Munirathinam, Mohd Farhan Arshi, Haleh Nazemi, Gian Carlo Antony Raj, Arezoo Emadi

**Affiliations:** E-Minds Laboratory, Electrical and Computer Engineering Department, University of Windsor, Windsor, ON N9B 3P4, Canada; muniratp@uwindsor.ca (P.M.); arshi@uwindsor.ca (M.F.A.); haleh.nazemi@uwindsor.ca (H.N.); antonyrg@uwindsor.ca (G.C.A.R.)

**Keywords:** volatile organic compounds, multi-sensor array, virtual sensor array, hybrid sensor array, fringing field capacitance, polymer-based sensors

## Abstract

Detecting volatile organic compounds (VOCs) is essential for health, environmental protection, and industrial safety. VOCs contribute to air pollution, pose health risks, and can indicate leaks or contamination in industries. Applications include air quality monitoring, disease diagnosis, and food safety. This paper focuses on polymer-based hybrid sensor arrays (HSAs) utilizing interdigitated electrode (IDE) geometries for VOC detection. Achieving high selectivity and sensitivity in gas sensing remains a challenge, particularly in complex environments. To address this, we propose HSAs as an innovative solution to enhance sensor performance. IDE-based sensors are designed and fabricated using the Polysilicon Multi-User MEMS process (PolyMUMPs). Experimental evaluations are performed by exposing sensors to VOCs under controlled conditions. Traditional multi-sensor arrays (MSAs) achieve 82% prediction accuracy, while virtual sensor arrays (VSAs) leveraging frequency dependence improve performance: PMMA-VSA and PVP-VSA predict compounds with 100% and 98% accuracy, respectively. The proposed HSA, integrating these VSAs, consistently achieves 100% accuracy in compound identification and concentration estimation, surpassing MSA and VSA performance. These findings demonstrate that proposed polymer-based HSAs and VSAs, particularly with advanced IDE geometries, significantly enhance selectivity and sensitivity, advancing e-Nose technology for more accurate and reliable VOC detection across diverse applications.

## 1. Introduction

Biomimetic based sensing technologies that are inspired by biological systems, have advanced rapidly in recent decades and found applications across diverse fields in healthcare [1], security [2], agriculture [3], transportation [4], energy production [5], consumer electronics [6], and education [7]. However, replicating the biological senses of smell (olfaction) and taste (gustation) remains challenging. This is due to the complex and dynamic nature of odors and flavors, which consist of diverse and often low-concentration chemical mixtures that are difficult to distinguish using conventional sensor technologies [8]. Despite these challenges, developing an effective olfactory system could provide a valuable tool for detecting chemical compounds beyond human visual and auditory perception.

Historically, animals with highly developed olfactory senses, such as canines, have been employed in critical applications like search and rescue, medical diagnostics, and agriculture. Dogs, for example, can detect human scents across vast and challenging terrains, identify diseases like cancer and diabetes through odor biomarkers, and recognize signs of infection and invasive pests in crops [9,10]. The widespread use of these biological detection systems highlights the need for artificial technologies that can match or even surpass natural olfactory capabilities [11].

Electronic noses (e-Noses) aim to replicate the capability to detect and analyze volatile organic compounds (VOCs) with high accuracy. These systems have the transformative potential in applications such as environmental monitoring, where they can detect harmful pollutants in air quality assessments, and healthcare, where they enable non-invasive diagnostics through odor signatures related to medical conditions [12]. The early detection of diseases like cancer and diabetes through breath analysis holds significant potential to enhance diagnostic accuracy and reduce the cost of healthcare [13].

Micromachined gas sensors have gained attention for e-Nose applications due to their small size, low power consumption, and high sensitivity. These characteristics enable the miniaturization and mass production of portable, cost-effective devices [14]. Interdigitated electrode (IDE)-based sensors provide advantages with regard to versatility, functionalization with various sensing materials, and adaptability for various detection mechanisms compared to other micromachined sensors [15].

Traditional multi-sensor arrays (MSAs) improve selectivity by employing multiple sensors functionalized with various polymers [15]. However, MSAs have limited performance as they rely mainly on direct chemicapacitive measurements without incorporating additional data. To address this, recent research introduced the concept of virtual sensor arrays (VSAs), which extract frequency-dependent data from a single sensor to improve selectivity [14,16]. While VSAs offer improved compound discrimination, they are typically limited to a single sensing material, which may restrict the diversity of chemical interactions they can capture. The present paper explores a novel approach that utilizes individual sensors as VSAs and integrates them into hybrid sensor arrays (HSAs).

In this paper, different IDE sensor topologies are designed, fabricated, and their performance is evaluated towards enhancing compound selectivity. HSAs encompass the strengths of MSAs as well as VSAs using integration of multiple physical sensors and employing Electrochemical Impedance Spectroscopy (EIS) based frequency-dependent responses to improve selectivity and sensitivity. This HSA-based approach helps to comprehensively analyze VOCs better than MSAs and VSAs for discriminating compounds. This study demonstrates improved performance of chemicapacitive sensors in VOC detection and has potential in environmental monitoring and industrial safety applications. Also, it presents solutions for reducing sensor count in e-Nose systems while ensuring high selectivity and sensitivity.

## 2. Operation Principle of Chemicapacitive Sensor

When a potential difference (*V*) is maintained between two adjacent conductor plates, they pick up opposite charges. The capacitance (*C*) of this configuration is directly proportional to the quantity of charge (*Q*) stored on the plates for the given voltage. Assuming vacuum as the medium between the plates, each of area (*A*), separated by a distance (*d*), the capacitance is given by [17]:

(1) $$C=\frac{Q}{V}=\frac{\varepsilon_{O}A}{d}$$

where *ε*_0_ is the permittivity of vacuum. When a dielectric is introduced as a medium between plates, the capacitance increases by a factor of the relative permittivity of the medium, *ε_m_* [14]:

(2) $$C=\frac{\varepsilon_{O}\varepsilon_{m}A}{d}$$

Equation (2) is derived considering that the electric field is confined in the medium between the two plates.

In a chemicapacitive sensor, the sensing layer between the electrode serves as a receptor that interacts with the target compounds. When these compounds are adsorbed, they alter the physical and chemical properties of the medium [17]. This interaction causes a shift in the material’s dielectric constant from *ɛ_m1_* to *ɛ_m2_*, leading to a measurable change in capacitance. The sensitivity of the sensor is (*S*_*C*_) defined as the change in capacitance (∆*C*) with respect to the change in target concentration (∆*M*), as given by Equation (3), applicable only for parallel plate configuration [14]:

(3) $$S_{C}=\frac{∆C}{∆M}=\frac{C_{2}-C_{1}}{M_{2}-M_{1}}=\frac{\frac{\varepsilon_{O}\varepsilon_{m2}A}{d}-\frac{\varepsilon_{o}\varepsilon_{m1}A}{d}}{M_{2}-M_{1}}=\left(\frac{A\varepsilon_{O}}{d}\right)\cdot \left(\frac{\varepsilon_{m2}-\varepsilon_{m1}}{M_{2}-M_{1}}\right)$$

**Figure 1:**
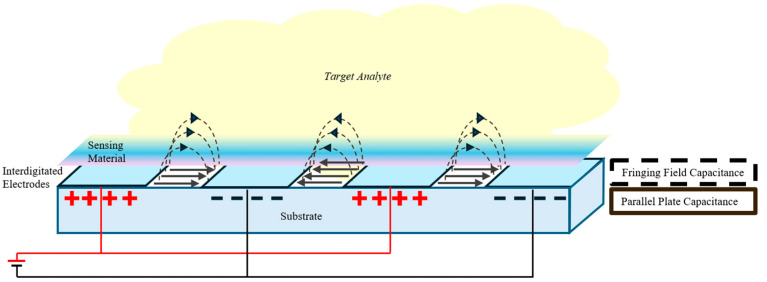
Schematic showing fringe field in a parallel plate capacitor.

The fringing fields which permeate the surrounding space apart from the dielectric material, as illustrated in Figure 1, cause the total area (*A*) to be effectively larger than the true area, resulting in a higher capacitance value [15,17].

Equivalently, the measured permittivity becomes larger than that of a parallel plate capacitor. Now summing both the parallel plate capacitance (*C*_*p*_) and the fringing field capacitance (*C*_*f*_), the total capacitance (*C*_*T*_) provides an estimate of the relationship between the capacitance and relative permittivity of the sensing material as given below. As it is complex to model the exact contribution of fringing field to the total electric field, it is either measured experimentally or estimated using Finite Element Analysis [14,18,19].

(4) $$C_{T}=C_{p}+C_{f}$$

(5) $$C_{T}\approx \frac{\varepsilon_{O}\varepsilon_{m}A}{d}$$

(6) $$S_{C}=\frac{∆C_{T}}{M}$$

In sensing applications, the upper plate of a parallel plate configuration is often perforated with holes to allow the target molecules to reach the dielectric sensing layer beneath it [20]. However, this design limits the effective sensing area. To overcome this, IDE configurations are proposed that involve coating one side of a series of electrodes. This allows the entire surface of the sensing material to be directly exposed to the compound or environment [21]. This direct exposure significantly improves the sensor’s response time compared to traditional parallel plate designs [21].

**Figure 2:**
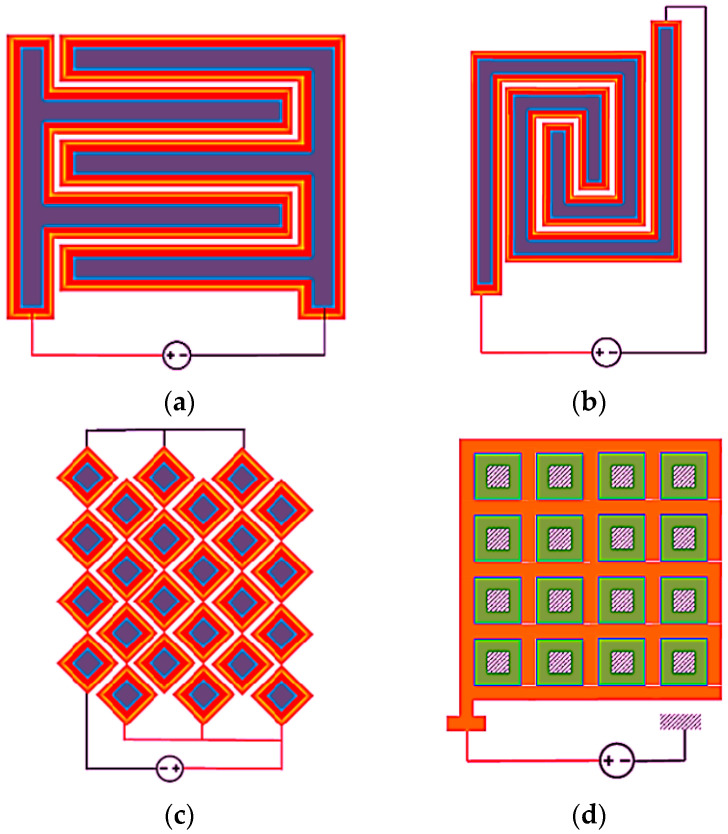
Schematic of four IDE fringe field geometries, such as (**a**) rectangular, (**b**) rectangular spiral, (**c**) frustum, and (**d**) grid.

Four different fringing field geometries are chosen in the present work based on their lesser complexity of fabrication complexity and high sensitivity [18]. The frustum and grid IDEs, as shown in Figure 2c and Figure 2d, respectively, have been designed for fabrication and testing. The rectangular and rectangular spiral geometries, as illustrated in Figure 2a,b, are fabricated using the PolyMUMPs process, which serve as baseline geometries for sensor validation. The PolyMUMPs fabrication process begins with a 300–500 µm-thick n-type silicon substrate coated with a doped phosphosilicate glass (PSG) layer to create a conductive surface [22]. A 600 nm silicon nitride layer is then deposited to electrically insulate the substrate. The process involves sequential deposition and patterning of three polysilicon layers (P0, P1, and P2), separated by sacrificial PSG oxide layers. Electrical and mechanical connections between these layers are established using anchor and via openings patterned via photolithography and etched with reactive ion etching (RIE) [22]. A 0.5 µm-thick gold layer is applied and patterned using a lift-off process for metallization. The final structures are released by immersing the dies in hydrofluoric acid to remove the sacrificial oxides, followed by rinsing and CO_2_ drying [22]. The IDEs are designed using the PolyMUMPs fabrication process to maximize the effective cross-sectional area of the parallel plate configuration. This is achieved by incorporating all three available polysilicon layers, resulting in a total electrode thickness of 4 µm [18,22]. To further enhance the sensing area, the number of electrode pairs per unit area is increased by minimizing the electrode width within the fabrication limits. The design maintained a 4 µm vertical gap between layers, with minimum feature sizes of 27 µm (P0), 19 µm (P1), and 11 µm (P2), and a lateral gap of 2 µm between adjacent electrodes [18,22]. These dimensions are consistently applied across all IDE configurations.

This study explores the use of chemicapacitive IDE VSAs to enhance selectivity, providing an alternative to the conventional MSA method. It also introduces HSAs as a novel approach to improve the selectivity of e-Nose systems through a novel operational strategy. An HSA consists of an MSA of chemicapacitive IDE sensors, each functionalized with a different sensing material. By operating each IDE in the array as an individual VSA, the sensor array effectively integrates both multi-sensor and virtual sensors. HSAs have the potential to record both the collective and complementary data from the sensor array, leveraging the strengths of both MSAs and VSAs, resulting in significantly improved selectivity and compound discrimination.

## 3. Experimental Setup

**Figure 3:**
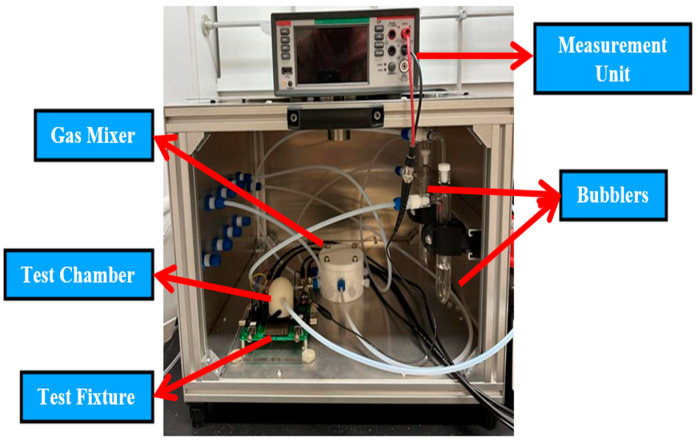
Photograph of experimental setup with sensor device, test fixture, FLOCON system, and measurement unit.

The sensor chip is placed within a sealed test chamber in a fume hood to ensure a controlled test environment required to compare the behavior of each gas sensor design. A Plasmionique FLO07-TSV flow control system is utilized to maintain the controlled test environment. Measurement systems include a Keithley DMM6500 for capacitive measurements and a Keysight 4990A impedance analyzer for conducting EIS on the chemicapacitive sensors that are being evaluated, as illustrated in Figure 3. A 5.0 ultra-high purity dry N_2_ (NI 5.0UH-T) is used as the carrier gas. To regulate and monitor the mass flow rate of the dry N_2_ into the test chamber, two out of the four Mass Flow Controllers (MFCs) available with the system are utilized, with one of them supplying the bubbler.

The sensors are coated with different sensing materials, particularly Polyvinylpyrrolidone (PVP) and Polymethyl methacrylate (PMMA) layers. The sensors are functionalized using the drop-casting method, selected for its simplicity and cost-effectiveness [18]. In this process, multiple drops of polymer solution are applied directly onto the interdigitated electrodes (IDEs) to ensure uniform coverage of the sensing area. The sensing materials included PVP with an average molecular weight of approximately 40,000 g/mol (Polysciences, Inc., catalog# 01052-250, Warrington, PA, USA) and PMMA with a viscosity-based molecular weight of approximately 75,000 g/mol (Polysciences, Inc., catalog# 01062-420). These sensors are positioned in the controlled test environment of the Plasmionique FLO07-TSV, which provides a stable platform for evaluating the HSA and selectivity of the resulting system. Three compounds, like relative humidity (RH), ethanol, and toluene, are selected for testing due to their relevance in environmental monitoring and greenhouse applications.

The initial step in assessing the behavior of MSA involves measuring its capacitance response. This array, comprising sensors functionalized with PMMA and PVP, establishes a baseline for evaluating the performance of VSAs and HSAs. The sensors are subjected to varying concentrations of RH, ethanol, and toluene, and the corresponding changes in capacitance (ΔC) are captured by the DMM6500. The measurement cycle is completed with the MSA to record the ΔC of the sensor array when exposed to various compounds and concentrations.

To enhance selectivity, EIS is utilized to initiate a transition from MSA to VSA. The EIS captures the sensor’s frequency response, providing detailed insights into both its capacitive and resistive properties across a wide frequency range. This frequency-dependent data reveals distinct patterns linked to different compounds, enhancing differentiation and selectivity. For this proof-of-concept, all EIS measurements are conducted under the same frequency sweep parameters. The frequency range is defined from 100 Hz to 10 MHz, which is the maximum frequency range of the E4990A impedance analyzer. The number of measurement points within this frequency range is fixed at 1602, enhancing the quantity of data collected per measurement. The 1602 points are distributed logarithmically across this entire frequency range, ensuring the uniform representation of data pertaining to each logarithmic scale. Therefore, at each EIS data point, the system recorded the magnitude of impedance (|Z|), phase angle (θ), resistance (R), reactance (X), and frequency (f), obtained at 1602 different frequencies between the range providing a rich dataset for analysis. All EIS measurements are carried out in a controlled environment using ultra-high-purity dry nitrogen (N_2_) as the carrier gas to eliminate humidity interference. For each analyte, ethanol, toluene, and relative humidity, exposure time is set to 30 min to allow the sensor response to stabilize across three concentration levels at 10%, 25%, and 50%. Since VSA includes only one physical sensor, two separate VSAs are implemented by PMMA and PVP, respectively. In HSA configuration, the output from both these sensors is combined, producing a comprehensive set of 3204 data points per measurement. This fusion of material diversity and frequency-domain information forms the foundation for the system’s enhanced selectivity and compound discrimination.

## 4. Results

### 4.1. Capacitance Measurements

**Figure 4:**
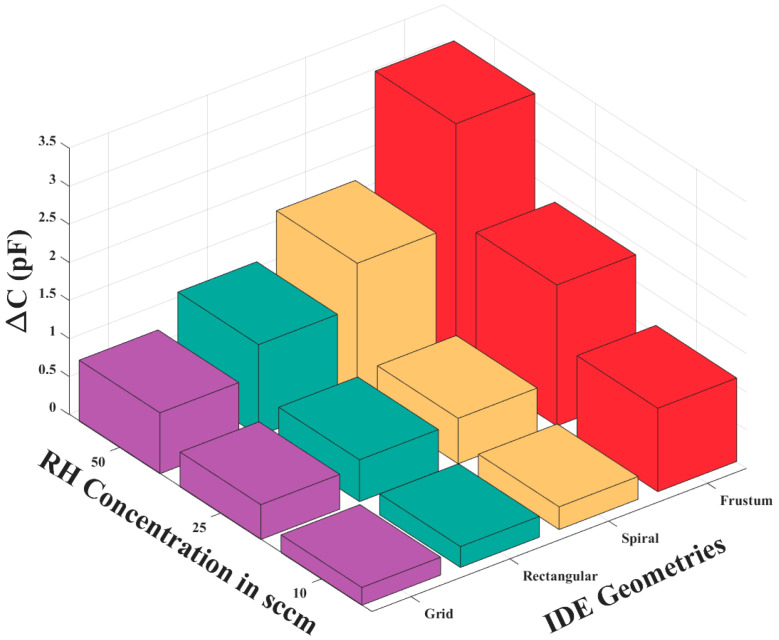
Change in capacitance vs. RH concentration for four sensor geometries.

To evaluate the repeatability of the sensor, a two-phase concentration cycling test was conducted using RH as the target analyte. In the first phase, RH levels were progressively increased from 0 to 10 to 25 to 50 SCCM. In the second phase, the concentration was decreased in reverse order from 50 to 25 to 10 to 0 SCCM. Throughout both cycles, the measured capacitance responses closely tracked the changes in RH concentration, with minimal variation between cycles. These results indicate that the sensor exhibited consistent behavior and negligible drift across repeated measurements, confirming its repeatability under controlled test conditions. The change in capacitance (ΔC) obtained for each IDE geometry at various RH concentrations is shown in Figure 4. The results show that the frustum IDE demonstrates the highest capacitive sensitivity compared to the other fabricated geometries in this proof-of-concept study. According to Randles’ circuit model, the capacitive reactance of the sensor is directly affected by its capacitive sensitivity [16]. Consequently, frustum IDE chemicapacitive sensors are chosen for further integration into the MSA, VSA, and HSA configurations.

**Figure 5:**
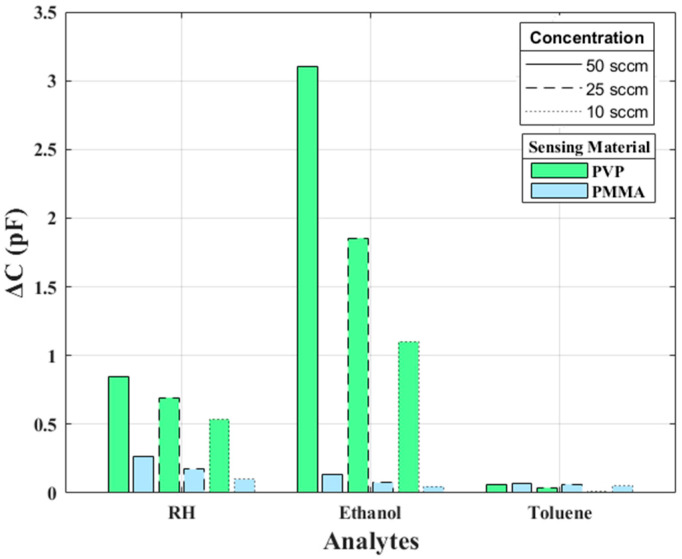
The capacitive response of MSA with PVP and PMMA-coated IDEs.

The MSA, which consists of two sensors, one coated with PMMA and the other with PVP, provides a pair of capacitance readings for each measurement. Figure 5 shows the data collected from the MSA. This shows the capacitance shift that occurs when the sensors are exposed to various compounds. However, these changes are largely non-specific, reflecting only whether the capacitance increases or decreases, without uniquely identifying the analyte. While the MSA shows some variation in response between compounds, there is considerable overlap in the data across the various sensor channels, which limits the system’s overall selectivity. This limitation underscores the need for an exploration of alternative configurations, such as the VSA.

### 4.2. EIS Measurements

**Figure 6:**
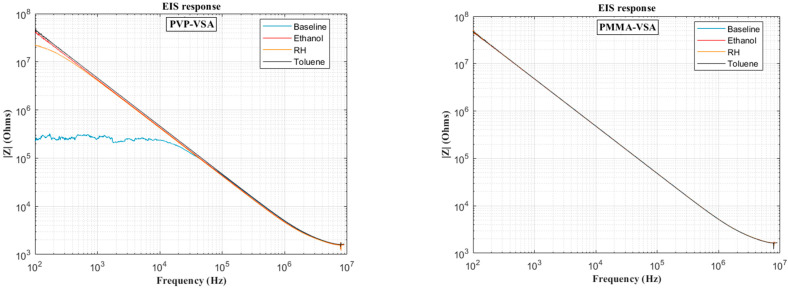
Plot of frequency versus impedance at 50 SCCM concentration of PVP (**left**) and PMMA (**right**) VSAs.

**Figure 7:**
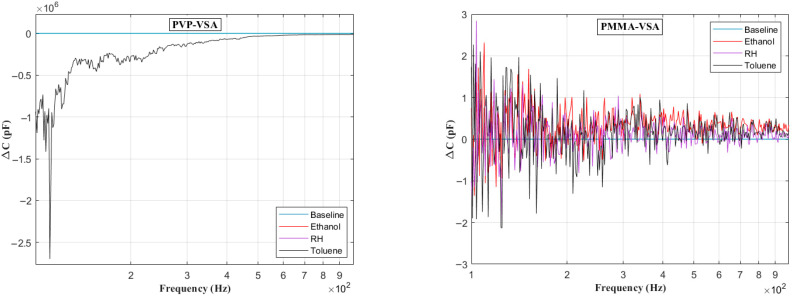
Change in capacitance vs. frequency plots in the range of 100 Hz and 1000 Hz for PVP (**left**) and PMMA (**right**) chemicapacitive VSAs.

**Figure 8:**
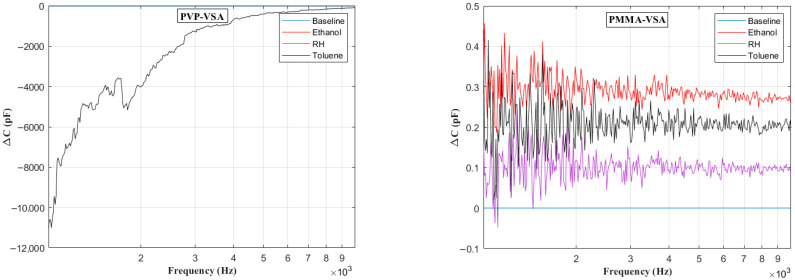
Change in capacitance vs. frequency plots in the range of 1 kHz to 10 kHz for PVP (**left**) and PMMA (**right**) chemicapacitive VSAs.

**Figure 9:**
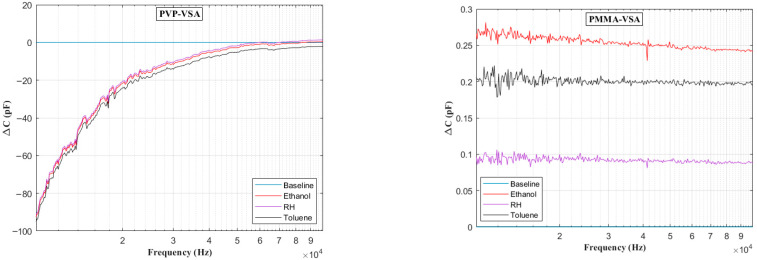
Change in capacitance vs. frequency plots in the range of 10–100 kHz for PVP (**left**) and PMMA (**right**) chemicapacitive VSAs.

**Figure 10:**
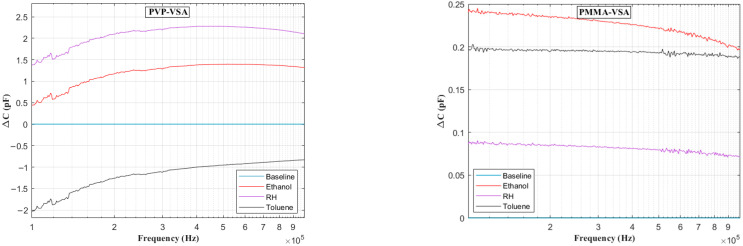
Change in capacitance vs. frequency plots in the range 100 kHz–1 MHz for PVP (**left**) and PMMA (**right**) chemicapacitive VSAs.

**Figure 11:**
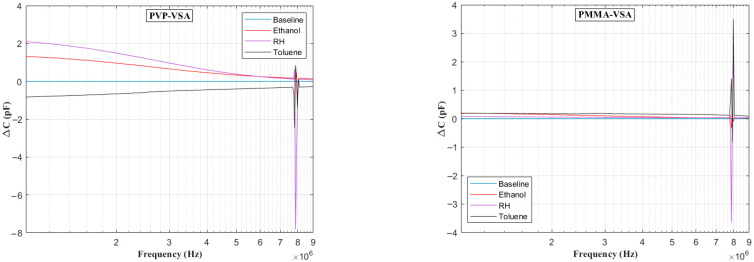
Change in capacitance vs. frequency plots in the range of 1–10 MHz for PVP (**left**) and PMMA (**right**) chemicapacitive VSAs.

Figure 6 presents the impedance frequency response for the PVP-VSA and PMMA-VSA configurations, measured at a 50% compound concentration. The results show distinct frequency-dependent behavior for each sensor across the 100 Hz to 10 MHz range. Moreover, a small spike in the measurements is seen at the end of the sweep range for both compounds, attributable to the resonance arising from the utilized test fixture. Based on the above data, the ΔC can be calculated to obtain the frequency-dependent behavior of the sensor dielectric as a function of input voltage frequency. Figure 7, Figure 8, Figure 9, Figure 10 and Figure 11 illustrate these ΔC trends across segmented logarithmic frequency intervals, allowing for clearer visualization. The results highlight that the two polymers, PVP and PMMA, exhibit different dielectric responses depending on the frequency range.

The baseline curves shown from Figure 6, Figure 7, Figure 8, Figure 9, Figure 10 and Figure 11 represent the reference sensor responses under dry nitrogen (N_2_) flow, without the presence of any analytes. The smooth and stable nature of these baselines indicates a low-noise, interference-free environment, which is critical for reliable sensing measurements. In Figure 7 (left) and Figure 8 (left), although curves for ethanol and relative humidity (RH) are present, they closely overlap with the toluene response due to similar dielectric behavior in the lower frequency ranges, 100 Hz to 10 kHz. This overlap causes visual indistinction in the original plots. Notably, differences become more apparent at mid-to-high frequencies, 10 kHz to 1 MHz, where specific interactions between polymer dielectric properties and analyte characteristics emerge, supporting the effectiveness of the EIS-based VSA and HSA configurations in enhancing selectivity.

### 4.3. Selectivity Analysis of Sensor Arrays

Selectivity refers to a sensor system’s ability to accurately identify a specific target gas within a given environment. A higher level of compound differentiation indicates that the sensor can better distinguish between various analytes, offering greater flexibility in detection. If the discrimination between two compounds is higher in one configuration as compared to another, the higher discriminating configuration exhibits higher selectivity at low concentration levels and encompasses a wider range of compounds. In sensor arrays, this also means that fewer sensors may be required to achieve the same level of selectivity as a lower-performing configuration.

The main objective of this work is to enhance the selectivity of the e-Nose system while minimizing the number of physical sensors needed to fabricate. To achieve this, the performance of the proposed HSAs and VSAs is evaluated based on their ability to distinguish between three target compounds in this proof-of-concept study. Scatter plots are a common approach for assessing the selectivity as they visually represent data points from the measurement system and help evaluate its ability to discriminate between different compounds. In this work, the scatter plots of the multivariate data recorded from both the VSA and HSA configurations are created by employing Principal Component Analysis (PCA). PCA reduces the dimensionality of complex datasets, making it easier to visualize patterns and differences in sensor responses. The generated data are further examined in the present work using a Linear Discriminant Analysis (LDA) model to emulate the e-Nose system. This is a supervised learning algorithm to differentiate between the compounds. LDA is chosen for its proven effectiveness in VOC selectivity and discrimination, as supported by previous studies [23]. In this study, all multivariate data analysis for the MSA, VSAs, and HSA is performed using MATLAB version 9.14.

### 4.4. EIS Data and Principal Component Analysis

As the number of features in a dataset increases, visualizing and interpreting the data becomes more challenging when it exceeds three dimensions. To address this, PCA is widely used in e-Nose applications for dimensionality reduction [14,23]. PCA is an unsupervised multivariate analysis technique that converts the data into a new coordinate system, where the principal components correspond to the directions of maximum variance in the dataset [23]. In this study, PCA is used to group the gas compounds based on similarities in their sensor responses, without requiring any prior knowledge about their identities. By emphasizing the principal components that capture the most significant components, PCA enhances the importance of the data along the principal directions. This leads to improving the visualization and interpretation of relationships within the dataset. These principal components serve as unique fingerprints or signatures, enabling the reliable detection and differentiation of individual compounds.

#### 4.4.1. PCA of PVP and PMMA VSAs

**Figure 12:**
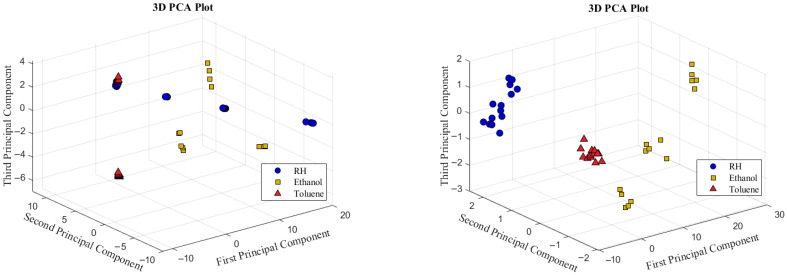
Three-dimensional representation of PCA for PVA (**left**) and PMMA (**right**) VSAs.

Prior to performing PCA on the multivariate EIS data, baseline normalization is applied to scale all capacitive reactance values between 0 and 1. The dataset for each VSA includes five measurements for each of the three target compounds at predefined concentrations, resulting in a total of 15 measurements per sensor configuration. Figure 12 shows the PCA plots of the first three principal components for both the PVP-VSA and PMMA-VSA, evaluated at compound concentrations of 10%, 25%, and 50% for the three compounds. These plots provide an informative representation of the data, revealing distinct clustering patterns that correspond to the different compounds. The distinct separation of these clusters highlights the effectiveness of the VSA configurations in accurately distinguishing between the compounds and their concentration levels.

#### 4.4.2. PCA of HSA

**Figure 13:**
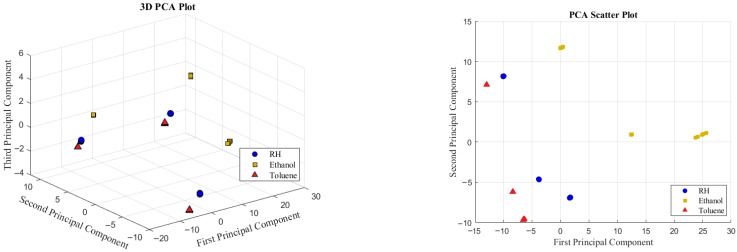
Three-dimensional representation of PCA for HSA (**left**) and its 2D projection along principal components 1 and 2 (**right**).

To implement the HSA configuration, the normalized EIS data of PMMA-VSA and PVP-VSA are concatenated to create a unified dataset. This merged dataset captures the frequency-dependent responses of both sensing materials and is then analyzed using PCA, as illustrated in Figure 13. As expected, the HSA effectively integrates the strengths of the individual VSAs, resulting in well-separated clusters in the PCA plot. These clusters show clear distinctions not only between different compounds but also among their concentration levels, thereby demonstrating the improved selectivity of HSA.

### 4.5. Explained Variance Analysis

Explained variance is a key statistical measure used in PCA to determine how much of the total variability in the dataset is captured by each principal component. It reflects how well the original data are represented when projected into a lower-dimensional space. With regard to sensor arrays, greater explained variance in the initial principal components suggests that a significant portion of the data variability is captured, which enhances the ability to distinguish between compounds. As such, explained variance is an important indicator of selectivity, as it shows how effectively a sensor array can distinguish compounds for different analytes. Table 1 summarizes the explained variance with the first three principal components (PCs) for three sensor array configurations: HSA, PVP-VSA, and PMMA-VSA. The explained variance assesses the proportion of total data variability captured by each principal component, which is related to the sensor array’s selectivity.

The PMMA-VSA configuration shows that its first principal component (PC1) captures a significant 90.2% of the total data variance. This indicates that most of the variation in sensor responses is concentrated along a single dimension. While this reflects a strong correlation in the sensor’s response to a particular compound or condition, it also suggests a finite contribution from the subsequent components, with PC2 capturing only 2.1% of the variance. As a result, PMMA-VSA may have limited selectivity, since it does not capture enough additional detail to effectively distinguish between multiple compounds. In contrast, the PVP-VSA configuration demonstrates a more balanced distribution of variance, with PC1 explaining 76.0% of the variance, PC2 capturing 19.8%, and PC3 contributing 3.3%. Although PC1 still captures the majority of the variability, the increased contribution of PC2 and PC3 suggests that the PVP-VSA is better equipped to capture distinct features from the target compounds, leading to improved selectivity. The HSA configuration demonstrates the most evenly distributed variance across the first three principal components, with PC1 explaining 64.9%, PC2 25.5%, and PC3 3.9%. This broader spread shows that HSA captures a wider range of variability, making it more capable of distinguishing between compounds. By combining the complementary characteristics of both PVP and PMMA sensors, the HSA achieves the highest level of selectivity. This makes it the most robust solution for compound discrimination, particularly in complex environments.

### 4.6. Linear Discriminant Analysis

To fully replicate the functionality of an e-Nose system, the sensor data are analyzed using a supervised machine learning algorithm, LDA. LDA is used to classify data into categories, such as compound type or concentration level, based on continuous sensor inputs. In this study, the continuous inputs comprise the preprocessed sensor responses (ΔC values for MSA and principal components for VSAs and HSAs), while the categorical variables correspond to the compound names and their concentrations. To ensure consistency across all sensor configurations, each dataset contains five measurements for each of three compounds at three different concentration levels, resulting in a total of 45 data points per configuration. LDA requires a training phase in which it learns to associate sensor responses with specific compound labels. After training, the model’s performance must be assessed on a dataset not used in the training set to evaluate the model’s generalization capability and avoid overfitting, where the model functions well on training data but imperfectly on unseen data. Given the limited dataset in this study, a cross-validation approach is used to estimate model performance more reliably. Specifically, Leave-One Out Cross-Validation (LOOCV) is employed as it increases the use of available data, which is especially advantageous in smaller datasets. In LOOCV, the model is trained on all data points except one, which is used as the test case. This process is repeated so that each data point serves as the test case exactly once. The final prediction accuracy is then calculated as the average accuracy across all iterations. Due to the different sensor data, with the MSA relying on capacitance changes and the VSAs and HSAs relying on frequency-dependent impedance characteristics, variations are introduced in the LDA training and testing process. In the case of the MSA configuration, the LDA model is trained using just two variables, one for the response from the PMMA sensor and one from the PVP sensor. For the HSA and VSA configurations, the number of input variables corresponds to the number of principal components selected for LDA training, ranging from one to five. The LOOCV prediction rate for the full dataset of 45 measurements, categorized by the number of variables used in the LDA training, is shown in Table 2. The prediction rate presented in Table 2 reflects the overall performance of the MSA, VSA, and HSA configuration when exposed to all three analytes, RH, ethanol, and toluene.

The LOOCV prediction analysis demonstrates that both the HSA and VSA configurations outperform the traditional MSA in terms of selectivity and classification accuracy. Notably, the HSA achieves 100% prediction accuracy when using four or more variables, highlighting its exceptional ability to distinguish between compounds. This can be attributed to the integration of PMMA and PVP responses, which takes a wider scale of compound-specific signals, thereby improving the system’s capability to differentiate between different compounds with high accuracy. Likewise, both VSA configurations significantly outperform the MSA. The PMMA-VSA reaches a 100% prediction rate with only two principal components, indicating its capability to achieve accurate classification with fewer variables. The PVP-VSA also performs well, with accuracy steadily increasing as more components are used, achieving a peak accuracy of 98% with five variables.

The VSA configurations significantly enhance system selectivity compared to the direct ΔC measurements from the MSA. For instance, when using only one variable, the PMMA-based chemicapacitive sensor in the MSA achieves a prediction rate of just 49%. In contrast, the corresponding PMMA-VSA reaches 77% under the same conditions and climbs to 100% accuracy with just two principal components. A similar pattern is observed with the PVP sensor, while the basic capacitive response yields an 89% prediction rate, the PVP-VSA improves this to 98% when five components are used. This comparison underscores the substantial advantage offered by the VSA approach that extracts a broader set of features related to the polymer–compound interactions by PCA. This enhancement is particularly beneficial in applications demanding highly selective compound discrimination, where VSAs deliver improved performance without the need for additional physical sensors. Further insights into the sensor-specific performance can be seen in their respective VSAs. The PMMA-VSA consistently exhibits high prediction accuracy, reaching a 100% prediction rate with only two principal components. This aligns with the explained variance distribution in Table 3, where PC1 accounts for over 90% of the total variance, making the inclusion of additional components less critical. In contrast, the PVP-VSA requires more principal components to reach its highest prediction accuracy, starting at 67% with one component and gradually increasing to 98% with five components. The HSA configuration, which integrates the responses from both the PMMA-VSA and PVP-VSA, achieves perfect prediction accuracy when four or more variables are used. This highlights the strength of combining data from multiple sensors by providing a more comprehensive view of the compounds. The system is better equipped to perform reliably under varying conditions. The HSA benefits from the complementary nature of the PMMA and PVP sensors. This integration translates into robust performance across varying compound types and concentrations. The nature of predictions made also contributes to the high accuracy of the PMMA sensor, as does that of the PVP sensor. Though the PMMA sensor reveals a lower overall response, it provides more consistent information in predicting the compound label regardless of the concentration. This difference in performance can be connected to the fact that the LDA model predicts the compound label without taking variations in concentration into account.

Hence, MSAs offer a straightforward approach by utilizing different sensing materials to improve selectivity; they are limited in their ability to distinguish between analytes with overlapping responses, particularly when operating based solely on static capacitance values [15,17]. VSAs, which incorporate frequency-dependent impedance spectroscopy, provide enhanced data dimensionality and enable better discrimination of VOCs [14,23]. However, VSAs are typically constrained to single-material sensing, limiting their capacity to capture a broader range of analyte interactions. The proposed HSA strategically integrates the strengths of both MSAs and VSAs by combining multiple sensing materials, PMMA and PVP, and leveraging their electrochemical impedance responses. This hybrid approach enables the system to capture compound-specific signatures across both material and frequency domains, significantly improving selectivity and prediction accuracy. The resulting HSA configuration consistently achieved 100% prediction accuracy in classification tasks, as shown in Table 2, outperforming standalone MSA and VSA configurations and demonstrating its robustness for complex VOC detection scenarios [14,23]. To contextualize the performance of the proposed HSA, a horizontal comparison with recent literature is included in Table 3.

To further assess prediction accuracy, the dataset of 45 measurements is divided based on either compound type or concentration level. This approach enables a more targeted evaluation of the LDA model’s performance. When grouped by compound, each subset includes 15 measurements, five for each of the three different concentrations. Here, the LOOCV prediction rate shows the capability of the LDA model to predict the concentration of the compound depending on sensor data, as shown in Table 4. Table 4 provides a compound-specific breakdown of the prediction rate using LOOCV.

Conversely, when the data are grouped by concentration, each subset again contains 15 measurements, representing five from each of the three compounds. Under this condition, the LOOCV prediction rate assesses the ability to anticipate the compound across various concentrations. Table 5 details the LOOCV prediction rate for the dataset filtered by the compound concentrations.

An analysis of the LOOCV prediction rates, both by compound and concentration, further reinforces the superior performance of the VSAs and HSA. Both the HSA and PMMA-VSA consistently achieve 100% prediction rate across all compounds and concentrations, underscoring their remarkable selectivity. Even the PVP-VSA, which demonstrates a comparatively lower prediction rate of 60% for toluene, still significantly outperforms the PVP chemicapacitive sensor, which records a 0% prediction rate for the same compound. This discrepancy highlights the limited sensitivity of the PVP sensor to toluene; however, the VSA still provides significant improvements over the raw capacitive shift data. This analysis also shows that selectivity remains high even at low compound concentrations. All configurations, including the MSA, perform well at 10% compound concentration, with most attaining a 100% prediction rate of the compound label.

The proposed HSA exhibits strong anti-interference capability, achieved by combining two distinct sensing materials, PMMA and PVP, with frequency-dependent EIS. This dual-domain strategy enables the system to effectively distinguish between compounds, even when individual sensor responses overlap. By leveraging the complementary sensing behaviors of PMMA and PVP, the HSA reduces cross-sensitivity and significantly improves selectivity. This is reflected in the high prediction accuracy obtained across different compound types and concentrations.

## 5. Conclusions

The research conducted in this paper systematically showed the potential of VSAs and HSAs to improve the selectivity of chemicapacitive IDE sensors. Through the implementation of innovative array configurations and improved IDE geometries, this study presents a novel approach that enables a single sensor to perform tasks traditionally requiring multiple sensors, thereby offering a more efficient solution for VOC detection. Experimental validations began with the assessment of various IDE geometries. Among the various IDE geometries tested, the frustum design demonstrated the highest capacitive sensitivity, showing a maximum capacitance change (ΔC) of 3.10 pF at 50% relative humidity, outperforming the other configurations. These results highlight the superior performance of the frustum IDE for VOC sensing applications and support its selection for further development in sensor array systems. The performance of different sensor array configurations is subsequently evaluated using PMMA and PVP in MSA, VSA, and HSA configurations. The MSA configuration, which utilizes responses from PMMA and PVP-coated sensors, realized a prediction rate of 82% with two sensor responses. Whereas, the VSA and HSA configurations used PCA for the dimensionality reduction of the EIS data, thereby facilitating more effective feature extraction. The PMMA-VSA showed a 100% prediction rate with only two principal components, while the PVP-VSA achieved a 98% prediction rate using five components. These results underscore the effectiveness of VSAs in improving data analysis and thereby enhancing the capability of the system for compound discrimination, thus minimizing the number of physical sensors required in e-Nose systems. The HSA configuration further enhanced selectivity by integrating the EIS responses from PMMA and PVP sensors, leveraging the complementary nature of the data acquired. The HSA consistently showed a 100% prediction rate with four or more variables, surpassing the individual VSAs and the MSA. This highlights the advantage of combining sensor responses to enhance compound differentiation, particularly in complex sensing environments. Overall, the findings of this study demonstrate that the selectivity and sensitivity of eNose systems, especially those using chemicapacitive IDE sensors, can be significantly enhanced through strategic design and operating strategies. Specifically, improved fringing field geometries have been shown to boost both capacitive response and impedance sensitivity. The use of Virtual Sensor Arrays (VSAs) proved particularly effective in enhancing selectivity, enabling a single sensor to generate richer datasets. This reduces the need for large physical sensor arrays, offering a more efficient and scalable approach for eNose systems. These findings have potential advantages in areas that demand precise and sensitive compound detection and provide a robust platform for further development, bridging the gap between sensor design, material science, and practical application.

## Figures and Tables

**Table 1:** Array configurations and explained variance of three PCs.

Array Type	Explained Variance PC1 (%)	Explained Variance PC2 (%)	Explained Variance PC3 (%)
HSA	64.9	25.5	3.9
PVP-VSA	76.0	19.8	3.3
PMMA-VSA	90.2	2.1	0.7

**Table 2:** LOOCV prediction rate of different types of arrays.

Array Type	Sensing Layer	Number of Variables Used for LDA Training				
		1	2	3	4	5
		LOOCV Prediction Rate (%)				
MSA	PMMA	49	82	-	-	-
	PVP	89				
VSA	PMMA	77	100	100	100	100
	PVP	67	69	78	91	98
HSA	PMMA	67	91	91	100	100
	PVP					

**Table 3:** Comparison of VOC sensing array performance reported across recent studies.

Reference	Approach	Materials Used	Features Used	Reported Prediction Accuracy	Number of Sensing Materials
[15]	Traditional MSA	Multiple polymers	Static capacitance	~82%	≥2
[14]	VSA (IDE sensor)	Single polymer	Fringing field capacitance (EIS)	Up to 95%	1
[16]	VSA (MXene-based)	Single MXene sensor	Frequency-dependent impedance	~95–97%	1
Present work	HSA	PMMA + PVP	Combined EIS + multi-material	100%	2

**Table 4:** LOOCV prediction rate for subsets of data separated by compound.

Compound	Array Type	Sensing Layer	Number of Variables Utilized for LDA Training				
			1	2	3	4	5
			LOOCV Prediction Rate (%)				
RH	MSA	PMMA	100	100	-	-	-
		PVP	100				
	VSA	PMMA	100	100	100	100	100
		PVP	100	100	100	100	100
	HSA	PMMA	100	100	100	100	100
		PVP					
Ethanol	MSA	PMMA	93	80	-	-	-
		PVP	100				
	VSA	PMMA	100	100	100	100	100
		PVP	100	100	100	100	100
	HSA	PMMA	100	100	100	100	100
		PVP					
Toluene	MSA	PMMA	33				
		PVP	0	7	-	-	-
	VSA	PMMA	100	100	93	93	93
		PVP	0	27	53	60	60
	HSA	PMMA	100	100	100	100	100
		PVP					

**Table 5:** LOOCV prediction rate for subsets of data separated by concentration.

Compound Concentration (%)	Array Type	Sensing Layer	Number of Variables Utilized for LDA Training				
			1	2	3	4	5
			LOOCV Prediction Rate (%)				
10	MSA	PMMA	100	100	-	-	-
		PVP	100				
	VSA	PMMA	100	100	100	100	100
		PVP	100	100	100	100	100
	HSA	PMMA	100	100	100	100	100
		PVP					
25	MSA	PMMA	100	100	-	-	-
		PVP	80				
	VSA	PMMA	100	100	100	100	100
		PVP	87	100	100	100	100
	HSA	PMMA	100	100	100	100	100
		PVP					
50	MSA	PMMA	100				
		PVP	100	100	-	-	-
	VSA	PMMA	100	100	100	100	100
		PVP	100	100	100	100	100
	HSA	PMMA	100	100	100	100	100
		PVP					

## Data Availability

Data are contained within the article.
